# Psychiatric Adverse Events Associated With Infliximab: A Cohort Study From the French Nationwide Discharge Abstract Database

**DOI:** 10.3389/fphar.2020.00513

**Published:** 2020-04-22

**Authors:** Eve-Marie Thillard, Sophie Gautier, Evgeniya Babykina, Louise Carton, Ali Amad, Guillaume Bouzillé, Jean-Baptiste Beuscart, Emmanuel Chazard

**Affiliations:** ^1^ Univ. Lille, CHU Lille, ULR 2694, CERIM, Public Health Department, Lille, France; ^2^ Univ. Lille, Inserm, CHU Lille, UMR-S1172, Center for Pharmacovigilance, Lille, France; ^3^ Univ. Lille, Inserm, CHU Lille, UMR_S1172, Medical Pharmacology Department, Lille, France; ^4^ Univ. Lille, Inserm, CHU Lille, U1172 - LilNCog - Lille Neuroscience & Cognition, Lille, France; ^5^ University of Rennes, Inserm, CHU Rennes, UMR 1099 - LTSI, Rennes, France

**Keywords:** infliximab, adverse events, psychiatry, depression, database, pharmacoepidemiology, pharmacovigilance

## Abstract

**Introduction:**

Infliximab (IFX) was the first anti-tumor necrosis factor (TNFα) antibody to be used in the treatment of severe chronic inflammatory diseases, such as Crohn’s disease and rheumatoid arthritis. A number of serious adverse drug reactions are known to be associated with IFX use; they include infections, malignancies, and injection site reactions. Although a few case reports have described potential psychiatric adverse events (including suicide attempts and manic episodes), the latter are barely mentioned in IFX’s summary of product characteristics. The objective of the present retrospective study was to detect potential psychiatric adverse events associated with IFX treatment by analyzing a national discharge abstract database.

**Materials and Methods:**

We performed an historical cohort study by analyzing data from the French national hospital discharge abstract database (PMSI) between 2008 and 2014. All patients admitted with one of the five diseases treated with IFX were included.

**Results:**

Of the 325,319 patients included in the study, 7,600 had been treated with IFX. The proportion of hospital admissions for one or more psychiatric events was higher among IFX-exposed patients (750 out of 7,600; 9.87%) than among non-exposed patients (17,456 out of 317,719; 5.49%). After taking account of potential confounders in the cohort as a whole, a semi-parametric Cox regression analysis gave an overall hazard ratio (HR) [95% confidence interval] (CI) of 4.5 [3.95; 5.13] for a hospital admission with a psychiatric adverse event during treatment with IFX. The HR (95%CI) for a depressive disorder was 4.97 (7.35; 6.68). Even higher risks were observed for certain pairs of adverse events and underlying pathologies: psychotic disorders in patients treated for ulcerative colitis (HR = 5.43 [2.01; 14.6]), manic episodes in patients treated for severe psoriasis (HR = 12.6 [4.65; 34.2]), and suicide attempts in patients treated for rheumatoid arthritis (HR = 4.45 [1.11; 17.9]).

**Discussion:**

The present retrospective, observational study confirmed that IFX treatment is associated with an elevated risk of psychiatric adverse events. Depending on the disease treated, physicians should be aware of these potential adverse events.

## Introduction

Infliximab (IFX) is a chimeric monoclonal antibody that specifically binds to and neutralizes the inflammatory and immune mediator tumor necrosis factor alpha (TNFα). The antibody has been approved since 1999 for the treatment of chronic inflammatory diseases, such as rheumatoid arthritis, Crohn’s disease, ankylosing spondylitis, psoriatic arthritis, ulcerative colitis, and plaque. Its use is limited to adults with moderately to severely active disease and who have an inadequate response to conventional therapy.

A dose of 3 to 5 mg/kg of IFX is usually infused every two months. In France, IFX is administered exclusively by medical staff in a hospital environment, and around 730,000 vials are delivered each year [Haute autorité de santé (HAS), 2016]. Even though IFX’s efficacy is well established, some aspects of the drug’s safety profile are still being investigated. The most serious adverse reactions described in detail to date have been infections, malignancies, and injection site reactions (Colombel et al., 2004; Zabana et al., 2010). A few case reports have described suicide attempts (Roblin et al., 2006; Eshuis et al., 2010) and manic episodes (Elisa and Beny, 2010; Austin and Tan, 2012) associated with the use of IFX or other TNFα inhibitors (Kaufman, 2005; Ghossoub et al., 2016; Jafri and Sammut, 2018). Although these psychiatric adverse events have also been reported in other types of study (Quartier et al., 2003; Mendes et al., 2013; Mendes et al., 2014; Lafay-Chebassier et al., 2015; Pastore et al., 2018; Smith et al., 2018), they are barely mentioned in each TNFα-inhibitor’s summary of product characteristics (SPC) [Agence Nationale de sécurité du médicament et des produits de santé (ANSM), 2020]. According to the SPC for IFX, depression and insomnia are frequent, amnesia, agitation, confusion, drowsiness, and nervousness are uncommon, and apathy is rare [Agence Nationale de sécurité du médicament et des produits de santé (ANSM), 2020]. In contrast, some studies have suggested that IFX treatment is associated with better quality of life in patients suffering from treatment-resistant mood disorders (Soczynska et al., 2009). The characterization of these psychiatric adverse events is therefore essential for adequately assessing the risk-benefit ratio and improving the management of these events when they occur.

Over the last few decades, the systematic collection of inpatient data (notably as part of health payment systems) has enabled the implementation of large-scale, retrospective, observational analyses of administrative medical databases. These databases contain “big data” (Baro et al., 2015), and their analysis raises a number of new methodological issues (Chazard et al., 2018). In France, IFX is obligatorily administered during an inpatient stay, and so is recorded in hospital databases. Moreover, most serious psychiatric events also lead to hospital admission, and so are tracked in the same databases.

Hence, the objective of the present retrospective analysis of the French national hospital discharge database was to detect potential psychiatric effects of IFX therapy (including manic episodes, depressive disorders, suicide attempts, and psychotic disorders) leading to hospital admission, while taking account of potential confounders (such as the underlying disease).

## Materials and Methods

### Data Source

We performed a historic cohort study by reusing data contained in the French national hospital discharge database (*Programme de Médicalisation des Systèmes d’Information*, PMSI). The PMSI database contains standardized discharge reports for all inpatient admissions to nonprofit or for-profit general acute care and psychiatric hospitals in France. Around 29 million inpatient stays are recorded each year. Each discharge report contains the following information: the primary and associated diagnoses coded according to the International Classification of Diseases, 10^th^ revision (ICD-10) (WHO, 2020), the therapeutic and diagnostic procedures coded according to the French *Classification commune des Actes Médicaux* (CCAM) terminology [L'Assurance Maladie (Ameli), 2020], and the dispensation of certain expensive drugs coded according to the French *Unité Commune de Dispensation* (UCD) classification (Unités communes de dispensation prises en charge en sus Publication ATIH). The data in the PMSI database is primarily collected as part of France’s fee-for-service hospital funding system. Each patient aged 18 or over is given a unique, anonymous identifier, enabling all his/her inpatient stays across the country to be tracked. The present database analysis obtained approval from the French National Data Protection Commission (*Commission nationale de l’informatique et des libertés* (Paris, France); reference number: 2049035).

### Study Population

We first identified all patients aged from 18 to 99 admitted with a primary or associated diagnosis corresponding to an indication for IFX (namely rheumatoid arthritis, Crohn’s disease, ankylosing spondylitis, psoriatic arthritis, ulcerative colitis, and plaque psoriasis) between January 1, 2009, and December 31, 2014. The ICD-10 codes for these diagnoses are listed in **Table 1**. Next, we extracted all the patients’ inpatient stays between January 1, 2008, and December 31, 2014, in order to obtain at least one year of historical data before the study inclusion date. Hence, we excluded patients with a history of psychiatric disease at some point between January 1, 2008, and the study inclusion date. To that end, we searched for hospital admissions with the same ICD-10 codes as those used to definition the study outcomes (i.e., the psychiatric adverse events; see below and **Supplementary Table 1**). So as to include only newly exposed patients, we also excluded patients treated with IFX prior to their inclusion date. Since IFX administration is not recorded by for-profit hospitals, we excluded patients with at least one admission to a for-profit healthcare facility with a primary diagnosis corresponding to an indication for IFX (**Table 1**). For all patients, the follow-up period ended on December 31^st^, 2014, at the time of death, or when a psychiatric adverse event occurred.

**Table 1:** ICD-10 codes corresponding to indications for infliximab [according to (WHO, 2020)].

ICD-10 code	Diagnosis
K50*	Crohn’s disease [regional enteritis]
K51*	Ulcerative colitis
M05*	Rheumatoid arthritis with rheumatoid factor
M06*	Other rheumatoid arthritis
M45*	Ankylosing spondylitis
L40*	Psoriasis

*Denotes "followed by any character".

### Study Variables

#### Exposure

For each patient, we defined time sequences corresponding to exposure and time sequences corresponding to non-exposure. A period of exposure started with an administration of IFX (defined by the UCD code 9213713 for Remicade^®^, since IFX biosimilars were not on the market at the time of the study). In view of the pharmacokinetics IFX, we considered that exposure ended 10 weeks after the IFX administration (corresponding to seven terminal half-lives, after which time 99% of drug has been eliminated from the body) [Agence Nationale de sécurité du médicament et des produits de santé (ANSM), 2020]. Non-exposed sequences corresponded to all other periods.

#### Outcomes

We defined four outcomes of interest, interpreted as “a hospital admission with a diagnosis of …”): psychotic disorders, manic episodes, depressive disorders, and intentional self-harm (including suicide attempts). These outcomes correspond to 202 different ICD-10 codes selected and grouped by consensus between the investigators (**Supplementary Table 1**). Most of the codes come from ICD-10 chapters V (“Mental and Behavioral Disorders”) and XX (“External Causes of Morbidity and Mortality”). According to the French coding rules, some of those codes cannot be used as principal diagnoses; hence, we searched for the outcomes among both the principal diagnosis and any associated diagnoses for an inpatient stay. When a patient experienced more than one psychiatric outcome during the follow-up, only the first event was analyzed (since the risk of recurrence of psychiatric events is high (Gotlib and Hammen, 2010)).

#### Other Variables

The following variables were also extracted for each inpatient stay: age, sex, admission date, discharge date, length of stay, and in-hospital mortality. We considered four age classes: under 30 (the reference class), 30–49, 50–69, and ≥ 70. We also inferred the presence of comorbidities (such as cardiovascular, respiratory, neurological, rare, renal or liver diseases, diabetes, and cancer) by screening the ICD-10 codes used by the French state health insurance system [Caisse Nationale d'Assurance Maladie (CNAM), 2019].

### Statistical Analysis

We first performed a descriptive analysis of the variables of interest. Qualitative variables were quoted as the frequency (percentage). Quantitative variables were quoted as the mean ± standard deviation (SD) when normally distributed or as the median [interquartile range (IQR)] when non-normally distributed. The 95% confidence interval (CI) was calculated using a normal law. Independence between categorical variables was assessed using a chi-squared test or Fisher’s exact test, and difference between means of quantitative variables was tested using Student’s t-test or an analysis of variance. The occurrence of the outcomes was studied using a semi-parametric Cox regression model with time-changing covariates (Therneau and Gambsch 2000). We checked that the various tests’ validity conditions were satisfied. The following covariates were tested: IFX exposure as the time-dependent variable with multiple changes, the patients’ characteristics (age and sex), the disease of interest, and comorbidities. Cox models were built for each stratum: rheumatoid arthritis, Crohn’s disease, ankylosing spondylitis, psoriatic arthritis, ulcerative colitis, plaque psoriasis, and two or more indications in the same patient. The results were calculated as the hazard ratio (HR) (95CI%). Several sensitivity analyses were performed. Firstly, we tested the Cox model with a negative control for the CCAM code “AHPA009”, corresponding to carpal tunnel surgery. Secondly, we varied the exposure period from 4 to 28 weeks, according to the SPC [Agence Nationale de sécurité du médicament et des produits de santé (ANSM), 2020]. Lastly, as death is a competing event, we performed the analyses after excluding patients who had died in hospital. There were no missing data. All tests were two-sided, and the threshold for statistical significance was set to p < .05. Values below 1.10^-10^ were reported as “p=0”. All analyses was performed using R software (version 3.3.1) (R Development Core Team, 2011).

## Results

### Characteristics of the Study Population on Inclusion

**Figure 1:**
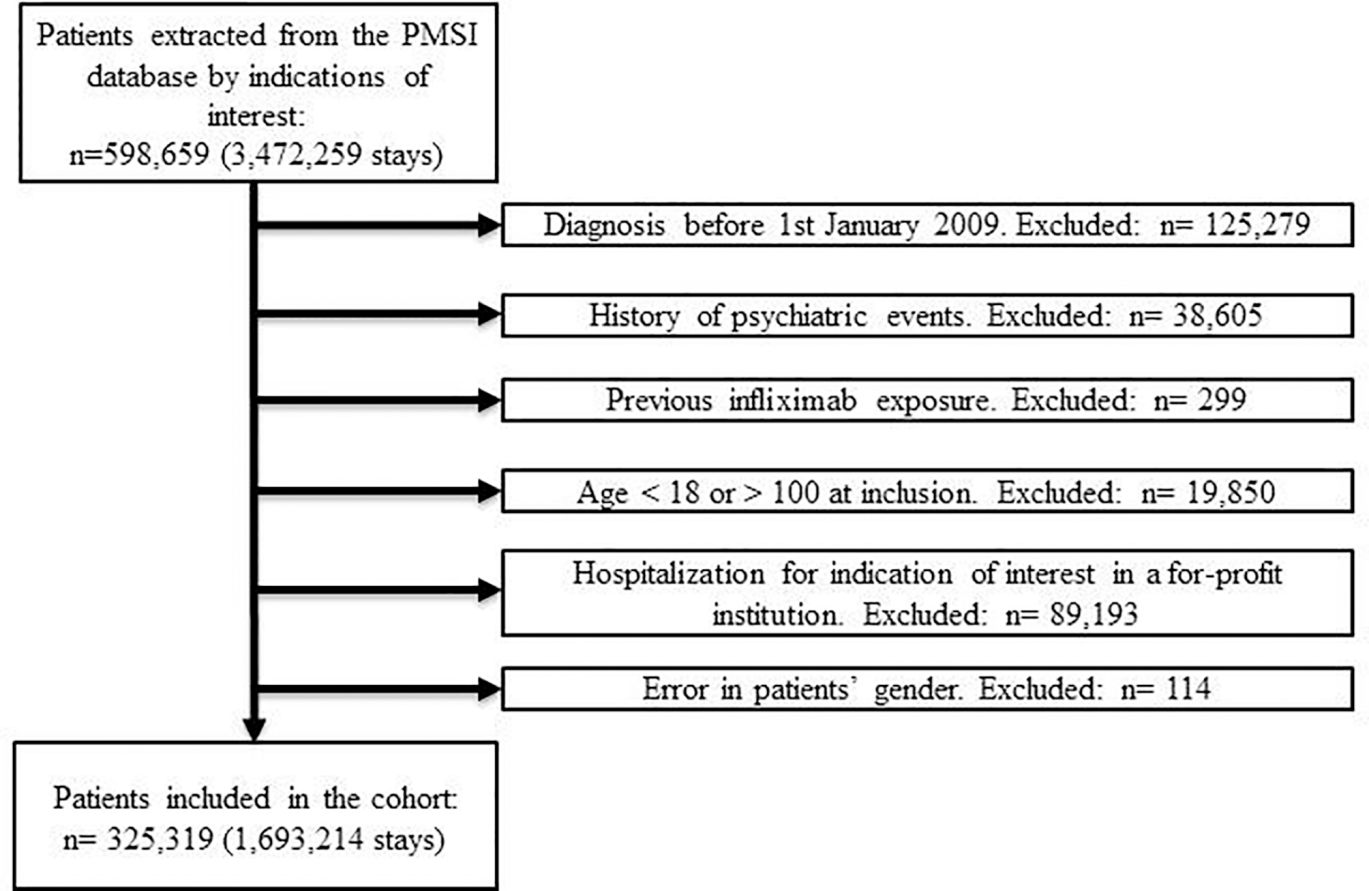
Flowchart.

Following the data extraction and then application of the exclusion criteria, the final cohort comprised 325,319 (54.3%) patients (**Figure 1**).

**Figure 2:**
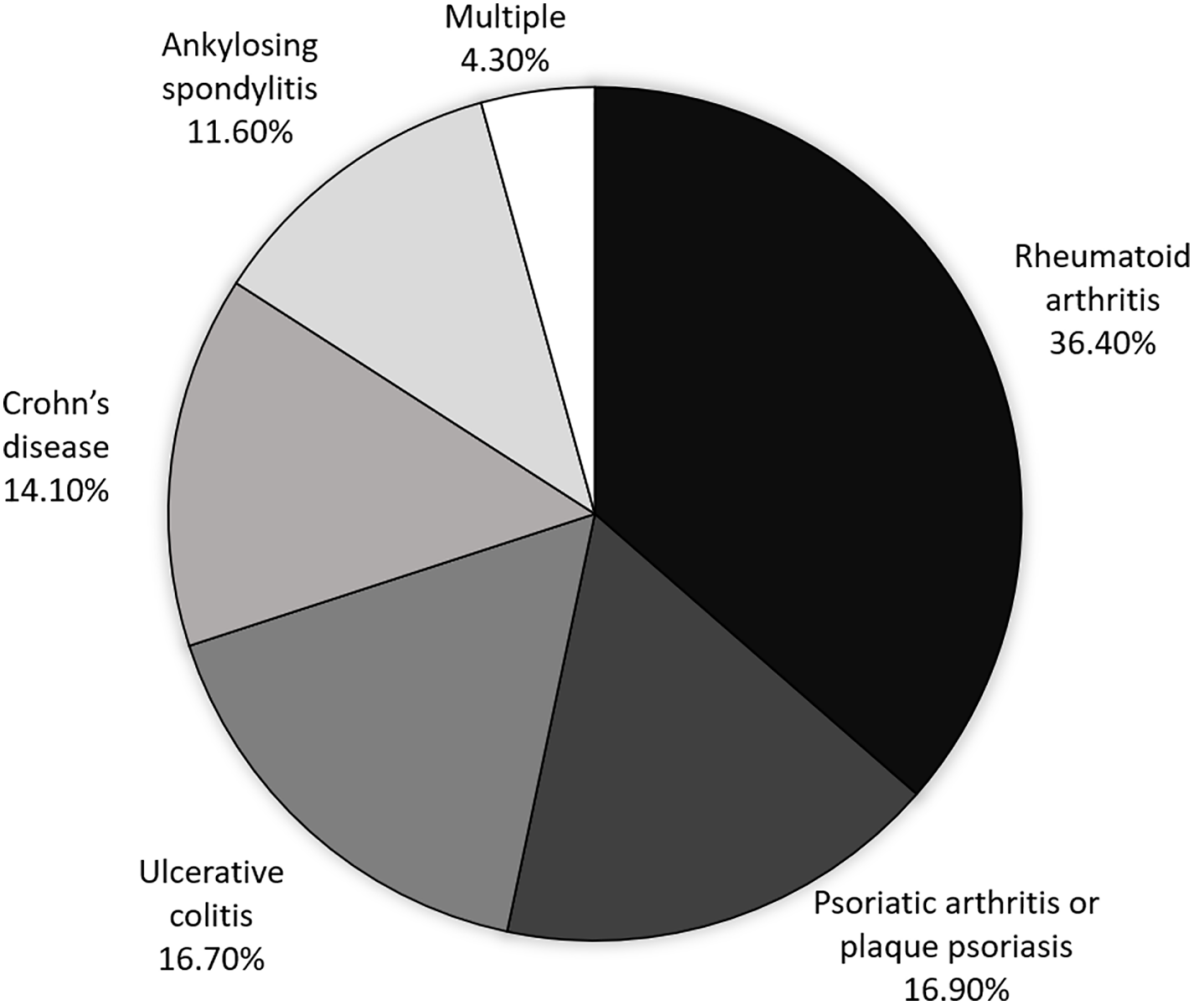
Infliximab indications in the cohort as a whole (n=325,319).

A total of 1,693,214 hospital stays by 325,319 different patients between January 1, 2008, and December 31, 2014, were included in the analysis. There were 182,796 (56%) females and 142,523 (44%) males, giving a sex ratio of 0.78. The mean ± SD age on inclusion was 58 ± 19. The median (IQR) number of hospital stays per patient was 4 (2;6), and the median (IQR) length of stay was 3 (1; 7) days. With regard to the disease of interest, 118,528 (36.4%) of the patients had rheumatoid arthritis, 54,982 (16.9%) had psoriatic arthritis or plaque psoriasis, 54,380 (16.7%) had ulcerative colitis, 45,674 (14.1%) had Crohn’s disease, 37,655(11.6%) had ankylosing spondylitis, and 14,100 (4.3%) had two or more of these diseases (**Figure 2**).

The patients’ characteristics are described by disease in **Table 2**.

**Table 2:** Characteristics of the study population by disease.

		Rheumatoid arthritis n=118,528	Psoriatic arthritis or plaque psoriasis n=54,982	Ulcerative colitis n=54,380	Crohn’s disease n=45,674	Ankylosing spondylitis n=37,655	Multiple indications n=14,100	All patients n=325,319
**At inclusion**	**Sex:** male, n(%)	35,304 (29.79)	32,306 (58.76)	28,263 (51.97)	20,243 (44.32)	20,131 (53.46)	6,276 (44.51)	**142,523 (43.81)**
	**Age class, n(%)**							
	18–29 years	3,217 (2.71)	3,248 (5.91)	5,752 (10.58)	11,529 (25.24)	4,607 (12.23)	1,973 (13.99)	**30,326 (9.32)**
	30–49 years	15,411 (13.00)	13,741 (24.99)	15,064 (27.70)	16,512 (36.15)	15,714 (41.73)	4,850 (34.40)	**81,292 (24.99)**
	50–69 years	41,595 (35.09)	22,783 (41.44)	19,683 (36.20)	11,699 (25.61)	12,259 (32.56)	4,918 (34.88)	**112,937 (34.71)**
	70–100 years	58,305 (49.19)	15,210 (27.66)	13,881 (25.53)	5,934 (12.99)	5,075 (13.48)	2,359 (16.73)	**100,764 (30.97)**
	**Patient with comorbidities, n(%)**	67,623 (57.05)	35,389 (64.36)	20,418 (37.55)	11,981 (26.23)	13,162 (34.95)	5,785 (41.03)	**154,358 (47.44)**
**At the end of the follow-up**	**Exposed to IFX, n(%)**	761 (0.64)	636 (1.16)	860 (1.58)	2,094 (4.58)	1,509 (4.01)	1,740 (12.34)	**7,600 (2.33)**
	**With a psychiatric event, n(%)**	7,798 (6.58)	3,281 (5.97)	2,106 (3.87)	1,903 (4.17)	1,677 (4.45)	1,441 (10.22)	**18,206 (5.60)**
	**With a psychotic disorder, n(%)**	1,766 (1.49)	789 (1.44)	514 (0.95)	371 (0.81)	276 (0.73)	215 (1.52)	**3,931 (1.20)**
	**With a manic episode, n(%)**	288 (0.24)	175 (0.32)	115 (0.21)	99 (0.22)	86 (0.23)	71 (0.50)	**834 (0.26)**
	**With a depressive disorder, n(%)**	6,492 (5.48)	2,686 (4.89)	1,713 (3.15)	1,573 (3.44)	1,456 (3.87)	1,290 (9.15)	**15,210 (4,68)**
	**With suicide attempts, n(%)**	385 (0.32)	270 (0.49)	186 (0.34)	248 (0.54)	206 (0.55)	121 (0.86)	**1,416 (0,44)**
	**Death, n(%)**	11,876 (10.02)	4,541 (8.26)	3,560 (6.55)	1,899 (4.16)	1,282 (3.40)	733 (5.20)	**23,891 (7.34)**

During the 6-year follow-up period, 7,600 patients (2.33%) were exposed to IFX, with a median 1 (1; 3) administrations per patient. This corresponded to 21,537 hospital admissions for an IFX infusion. The median time interval between successive perfusions was 54 (39; 63) days. The patients’ characteristics are summarized by drug exposure status in **Table 3**.

**Table 3:** Characteristics of the study population by infliximab exposure status at the end of the follow-up period.

		Exposed		Non-exposed		*p-value*
		n= 7,600		n= 317,719		
**At inclusion**	**Sex:** male, **n(%)**	3,624	(47.7)	138,899	(43.7)	*<0.001*
	**Age class, n(%)**					*0*
	18–29 years	2,074	(27.6)	28,252	(8.89)	
	30–49 years	3,115	(41.0)	78,177	(24.6)	
	50–69 years	2,007	(26.4)	110,930	(34.9)	
	70–100 years	404	(5.32)	100,360	(31.6)	
	**Disease, n(%)**					*0*
	Rheumatoid arthritis	761	(10.0)	117,767	(37.1)	
	Psoriatic arthritis or plaque psoriasis	636	(8.37)	54,346	(17.1)	
	Ulcerative colitis	860	(11.3)	53,520	(16.8)	
	Crohn’s disease	2,094	(27.6)	43,580	(13.7)	
	Ankylosing spondylitis	1,509	(19.9)	36,146	(11.4)	
	Multiple indications	1,740	(22.9)	12,360	(3.89)	
	**With comorbidities, n(%)**	1,850	(24.3)	152,508	(48.0)	*0*
**At the end of the follow-up**	**With a psychiatric event, n(%)**	750	(9.87)	17,456	(5.49)	*<0.001*
	**With a psychotic event, n(%)**	85	(1.12)	3,846	(1.21)	*0.501*
	**With a manic episode, n(%)**	40	(0.53)	794	(0.25)	*<0.001*
	**With depressive disorder, n(%)**	679	(8.93)	14.531	(4.57)	*<0.001*
	**With suicide attempts, n(%)**	85	(1.12)	1,331	(0.42)	*<0.001*
	**Death, n(%)**	123	(1.62)	23,768	(7,48)	*<0.001*

### Occurrence of Psychiatric Events

**Figure 3:**
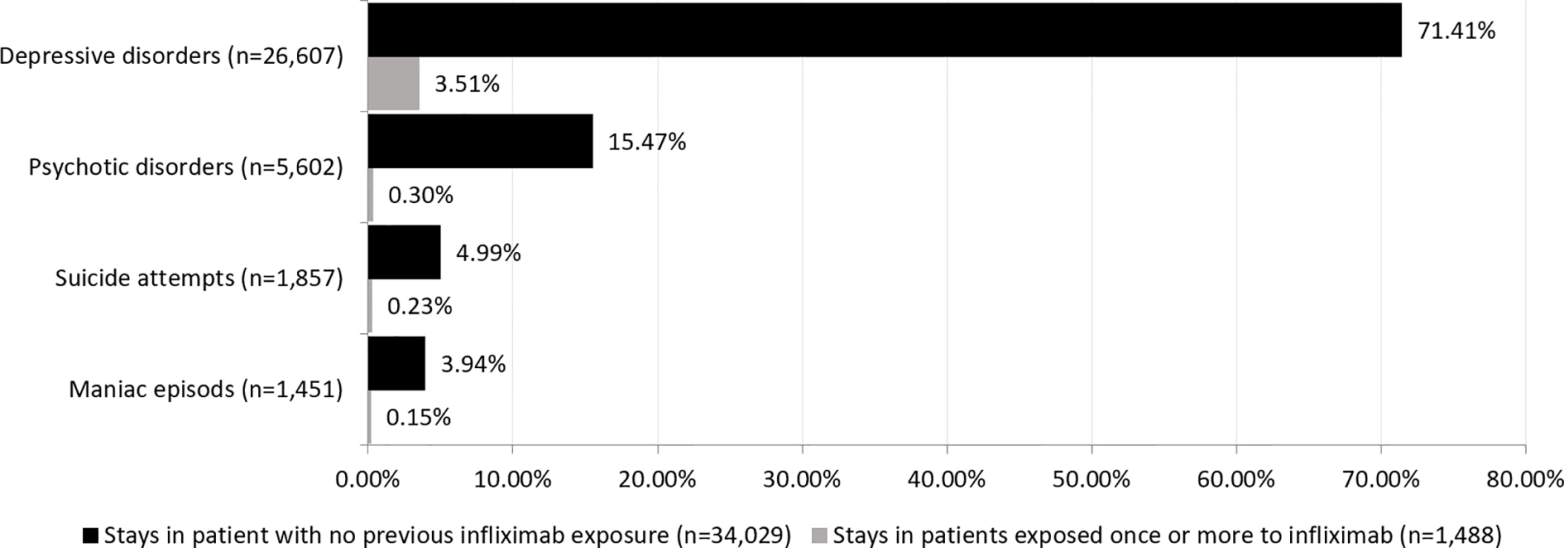
Types of psychiatric event (n=35,517) observed.

**Figure 4:**
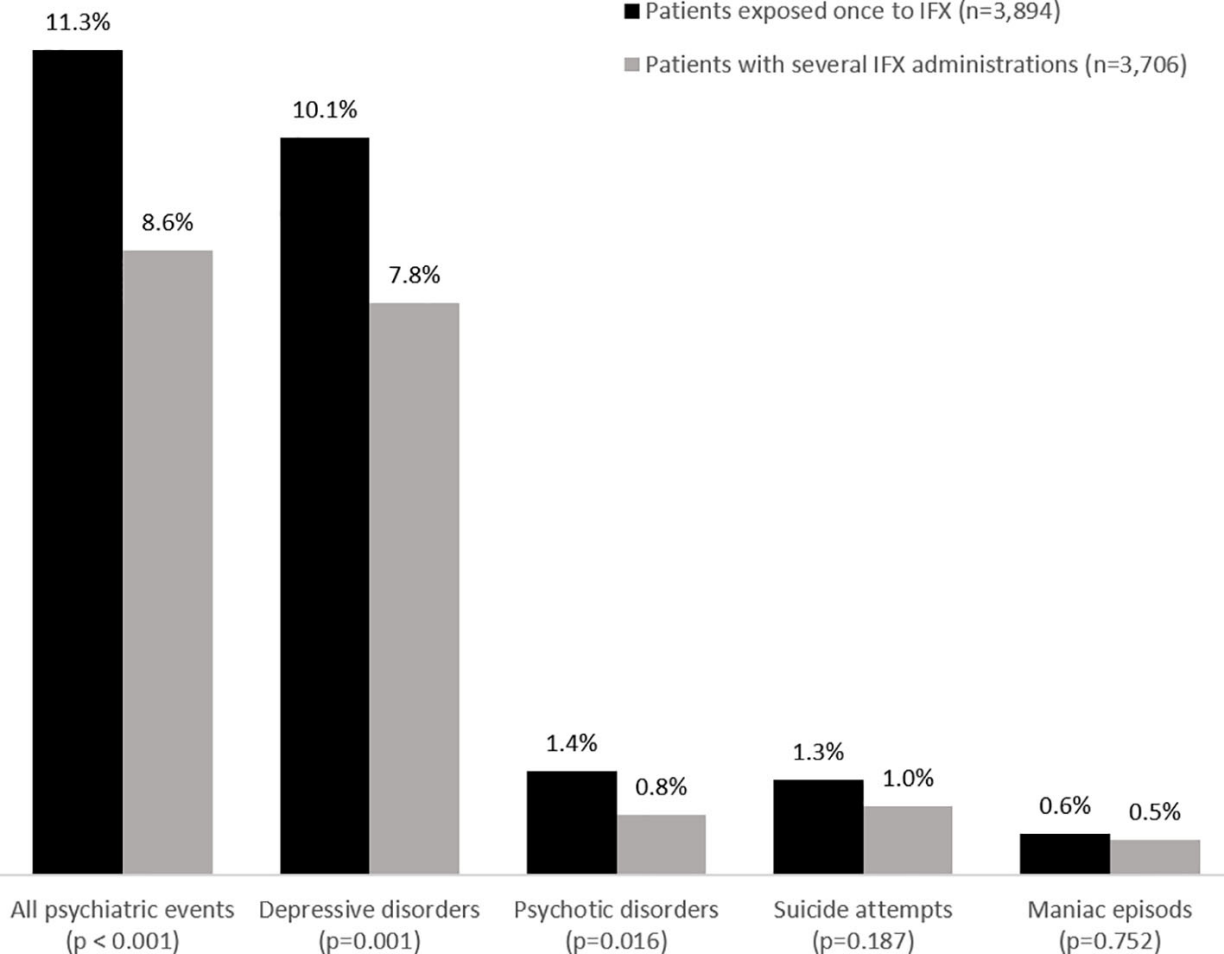
Psychiatric outcomes as a function of the number of prior infliximab administrations.

During the follow-up period, 32,102 (1.9%) hospital stays mentioned one or more psychiatric event as defined above (**Figure 3**). The stays concerned a total of 18,206 (5.6%) patients with one or more psychiatric events; 552 of these patients (3.03%) had been exposed to IFX before the occurrence of the first psychiatric event, with a median of 1 [1; 2] administrations. The distribution of psychiatric outcomes as a function of the number of IFX administrations is shown in **Figure 4**. The median time interval between the IFX administration and the occurrence of the psychiatric adverse event was 5 (2; 11) days.

After taking into account all the psychiatric outcomes for the entire cohort, we found that the following variables were significantly associated with a higher risk of experiencing a psychiatric event: sex, disease treated, IFX exposure, the presence of comorbidities (as defined in *Other Variables*), and age. The corresponding HR (95%CI) values are given in **Table 4**. With regard to IFX exposure, we found an overall HR (95%CI) of 4.5 (3.95; 5.13), i.e., a four-fold greater risk of hospitalization with a psychiatric disorder following IFX administration. The risk was higher still for a depressive disorder (HR [95%CI] = 4.97 [7.35; 6.68]). Higher risks were also observed for psychotic disorders in patients treated with IFX for ulcerative colitis (HR [95%CI] = 5.43 [2.01; 14.6]), manic episodes in patients treated for severe psoriasis (12.6 [4.65; 34.2]), and suicide attempts by patients treated for rheumatoid arthritis (4.45 [1.11; 17.9]). The HR in IFX-exposed patients are described by psychiatric event and by disease stratum in **Table 5**.

**Table 4:** Results of a Cox model regression for psychiatric events in the cohort as a whole (number of patients = 325,319).

	Hazard ratio (HR)	95% confidence interval
**Sex: male (ref = female)**	0.72	[0.70; 0.74]
**Disease (ref = Crohn’s disease)**		
Ankylosing spondylitis	1.06	[0.99; 1.13]
Ulcerative colitis	0.85	[0.80; 0.91]
Psoriatic arthritis or plaque psoriasis	1.20	[1.13; 1.27]
Rheumatoid arthritis	1.06	[1.01; 1.12]
Multiple indications	1.89	[1.76; 2.02]
**Infliximab exposure**	4.50	[3.95; 5.13]
**Comorbidities**	1.69	[1.64; 1.75]
**Age (ref = 18–29 years)**		
30–49 years	1.52	[1.41; 1.64]
50–69 years	1.61	[1.49; 1.73]
70–100 years	2.52	[2.34; 2.72]

**Table 5:** Hazard ratios (95%CIs) for infliximab exposure, as a function of the type of psychiatric event and the pathology treated.

	All outcomes	Psychotic disorders	Manic episodes	Depressive disorders	Suicide attempts
**All indications** **(325,196 patients)**	4.50 [3.95; 5.13] (18,112 events)	1.87 [1.12; 3.11] (3,894 events)	2.96 [1.52; 5.74] (827 events)	4.97 [4.35; 5.68] (15,136 events)	1.18 [0.59; 2.37] (1,397 events)
**Rheumatoid arthritis** **(118,528 patients)**	3.10 [1.92; 4.99] (7,762 events)	NC*	NC*	3.49 [2.17; 5.63] (6,464 events)	4.45 [1.11; 17.9] (379 events)
**Psoriatic arthritis or plaque psoriasis** **(54,982 patients)**	6.16 [4.39; 8.65] (3,262 events)	0.93 [0.13; 6.59] (781 events)	12.6 [4.65; 34.2] (172 events)	7.27 [5.18; 10.2] (2,674 events)	NC*
**Ulcerative colitis** **(54,380 patients)**	9.28 [6.52; 13.2] (2,095 events)	5.43 [2.01; 14.6] (509 events)	4.39 [0.61; 31.7] (114 events)	10.9 [7.57; 15.7] (1,701 events)	1.86 [0.26; 13.4] (185 events)
**Ankylosing spondylitis (37,655 patients)**	6.55 [4.97; 8.64] (1,668 events)	2.06 [0.51; 8.34] (270 events)	2.45 [0.34; 17.7] (84 events)	6.96 [5.23; 9.26] (1,448 events)	1.60 [0.40; 6.48] (203 events)
**Crohn’s disease** **(45,674 patients)**	3.04 [2.13; 4.34] (1,894 events)	1.59 [0.51; 4.98] (369 events)	NC*	3.37 [2.31; 4.90] (1,568 events)	0.60 [0.08; 4.26] (244 events)
**Multiple indications** **(14,100 patients)**	3.42 [2.67; 4.37] (1,431 events)	2.06 [0.84; 5.02] (211 events)	2.73 [0.85; 8.73] (71 events)	3.73 [2.91; 4.78] (1,281 events)	0.92 [0.23; 3.75] (120 events)

*NC, not computed. When no events were observed during exposed sequences, the 95%CI could not be computed.

During the follow-up period, 23,891 (7.3%) patients died in hospital.

All the sensitivity analysis gave similar results. The negative control (carpal tunnel surgery) confirmed the absence of excess risk, with an HR (95%CI) of 0.567 (0.212; 1.51).

## Discussion

### Main Results

The present study included 325,319 patients with diseases corresponding to specific IFX indications, and 7,600 of these individuals (2.33%) had been treated at least once with IFX. Among the IFX-exposed patients, 750 (9.87%) presented at least one psychiatric event; this proportion was 5.49% for non-exposed patients. The adjusted HR (95%CI) was 4.5 (3.95; 5.13) for psychiatric events and 4.97 (4.35; 5.68) for depressive disorder. These results took account of potential confounders, and were confirmed in sensitivity analyses. Depending on the disease in question, the risk of a psychiatric events was 3 to 9 times higher in IFX-exposed patients than in non-exposed patients. Female sex, old age, and comorbidities were also risk factors.

### Comparison With the Literature Data

We found that 8.93% of the IFX-exposed patients had a major depressive disorder, in agreement with the values quoted in the drug’s SPC (“frequent: from 1 to 10%”) [Agence Nationale de sécurité du médicament et des produits de santé (ANSM), 2020]. However, the incidence of a suicide attempt among IFX-exposed patients was higher in our study (1.12%) than in the periodic safety update report on IFX (“very rare: less than 1/10,000”) (Eshuis et al., 2010).

Meta-analyses about IFX safety profile mainly report malignancies, infusion reactions, and infections but no psychiatric adverse events (Peyrin–Biroulet et al., 2008; Wiens et al., 2009; Kawalec et al., 2013; Lemos et al., 2014; Michaud et al., 2014). However, the present study results confirm the presence of a signal suggested by several case reports in which IFX use is suspected to induce psychiatric disorders. Two patients treated for Crohn’s disease and ulcerative colitis attempt suicide after repeated IFX injections (Roblin et al., 2006; Eshuis et al., 2010). Manic symptoms (euphoria, insomnia, agitation…) appeared in two others patients after their first IFX administration, for one of them symptoms reappeared after 6 injections (Elisa and Beny, 2010; Austin and Tan, 2012). In the Portugese pharmacovigilance database, three cases of psychiatric disorders with no more details were reported (Mendes et al., 2013; Mendes et al., 2014). In an efficacy study about IFX and biosimilars, 10% of the 94 patients experience various adverse effects including depression (Razanskaite et al., 2017). In a safety study, a patient committed suicide four months after his last IFX infusion (Fidder et al., 2009).

It is well known that pathologies like inflammatory bowel disease, psoriasis, and rheumatoid arthritis are associated with psychiatric disorders in general and depression in particular (Dickens et al., 2002; Bouguéon and Misery, 2008; Graff et al., 2009; Olivier et al., 2010); we took this into account by stratifying our data. It is nevertheless noteworthy that in some studies, IFX has been found to relieve the symptoms of psychiatric disorders. For example, one case report featured the remission of psychotic symptoms in a patient suffering from Crohn’s disease (Reimer et al., 2009). Likewise, Ertenli et al. suggested that IFX treatment could improve quality of life by relieving the depression symptoms associated with ankylosing spondylitis (Ertenli et al., 2012). In their literature review, Soczynska et al. concluded that IFX treatment had a beneficial effect on the symptoms of bipolar disorder (Soczynska et al., 2009). These beneficial effects can be explained by (i) the pathologic elevation of blood interleukin-6 and C-reactive protein levels in psychotic disorders (Delaney et al., 2019), and (ii) IFX’s ability to reduce these elevated levels (Brietzke and Kapczinski, 2008; Brietzke et al., 2009). An antidepressant effect has also been suggested for other TNFα inhibitors, such as etanercept (Brymer et al., 2018).

With regard to depression and the associated risk factors, it is well known that women are twice as likely to suffer from major depression as men are. Moreover, older people are also more likely to suffer from depression (Gotlib and Hammen, 2010). Our results (an HR of 1.39 for women and 2.52 for patients aged 70 and over) are in line with these observations.

### Strengths of the Study

The present study’s main strength was its creation of a large historical cohort from a national database. To the best of our knowledge, our study is the first to have included such a large number of IFX-treated patients. We were therefore able to screen for rare adverse events. Moreover, most studies in the literature were limited to a single disease, whereas the present study covered all the indications for IFX approved in France. We used a control group of patients with the same diseases to eliminate the indication as a confounding factor, since it is well known that these diseases and/or their treatments are associated with psychiatric disorders (Dickens et al., 2002; Bouguéon and Misery, 2008; Graff et al., 2009; Olivier et al., 2010). Even though chronic comorbidities are not described in detail in the PMSI database, we took them into account as best we could; indeed, studies have shown that the prevalence of mental health problems (e.g., depressive disorders) among patients with a chronic disease (cardiovascular disease, diabetes, epilepsy, obstructive pulmonary disease, etc.) is higher than in the general population (Miorelli and Abe, 2016).

The reuse of data from reimbursement databases is limited by the fact that some patients do not actually take the medication that has been dispensed. Since IFX is exclusively administered in hospital in France, we considered that a record in the PMSI database is a reliable marker of exposure to the drug. Moreover, by excluding patients admitted to for-profit hospitals and by using the patients’ unique identifiers, we were able to establish each patient’s true timeline for IFX exposure with no missing values. Furthermore, by considering at least one year of data prior to the index IFX infusion, we could reasonably suppose that all patients included in the analysis were new IFX users. This exclusion criterion enabled us to avoid bias related to the depletion of susceptible individuals. We also limited bias by only considering the patient’s first recorded psychiatric adverse event, since psychiatric disorders like major depression have a high risk of recurrence (Gotlib and Hammen, 2010).

Our pharmacovigilance analysis of the PMSI database is an novel technique that has rarely been used to detection adverse events (Weill et al., 2010; Neumann et al., 2012; Heng et al., 2015). A recent report validated the use of the PMSI database for detecting serious adverse drug reactions (Osmont et al., 2013). Furthermore, the Cox regression models used in the present study are the most appropriate for survival analyses of medical data (Desquilbet and Meyer, 2005). A time-dependent variable allowed us to take account of changes in IFX’s exposure during patient follow-up. If the risk of an event persisted even after exposure had ended, this would have decreased the HRs calculated in the present study; hence, our estimates are conservative.

### Weaknesses of the Study

The major limitation of our study was its inability to take full account of some important confounding factors. Firstly, the PMSI database lacks information on the patient’s other drug prescriptions (usual treatments, self-medication etc.), which the exception of certain expensive treatments administered in hospital. This is a problem, give that IFX can be combined with methotrexate in some indications, and that corticoids are the first-line treatments for inflammatory diseases. Indeed, the SPC for methotrexate mentions the possible occurrence of psychiatric disorders such as depression and psychoses [Agence Nationale de sécurité du médicament et des produits de santé (ANSM), 2020], and the SPCs for corticosteroids also mention occasional manic episodes or depressive symptoms upon treatment discontinuation [Agence Nationale de sécurité du médicament et des produits de santé (ANSM), 2020]. These psychiatric symptoms are also reported in the scientific literature (Kenna et al., 2011). This limitation is partially taken into account by the stratification method used in the present study, since combination therapy is usually related to the disease being treated. Moreover, we were not able to determine whether or not our results were dose-dependent.

Secondly, we were not able to consider the severity of the disease treated with IFX. A difference in severity between exposed and non-exposed groups could result in an indication bias: the administration of IFX might then simply be a severity marker, with severity as the cause of the psychiatric event.

Thirdly, the PMSI database lacks data about the patient’s environment and background. It is well known that genetics and family history are risk factors for psychiatric disorders (Meyer-Lindenberg and Weinberger, 2006). Moreover, other factors can be associated with psychiatric conditions such as major depression; these include substance abuse (smoking, alcohol, drugs of abuse, etc.), educational level and socioeconomics conditions (Gotlib and Hammen, 2010; Kessler and Bromet, 2013).

A fourth limitation of our study is the relatively short (6-year) follow-up period for the detection of psychiatric conditions. In fact, psychiatric symptoms appear insidiously, and establishing a reliable diagnosis make years (François and Marie-Cardine, 2005).

We cannot rule out certain sources of bias, such as prescription bias, one can considered that psychiatric symptoms are be a sign of disease progression. Furthermore, patients treated with IFX (exclusively administered in hospital) are more likely to be hospitalized for psychiatric reasons than patients being managed primarily by their general practitioner (Berkson’s bias). The higher death rate in non-exposed patients could be explained by the efficacy of the IFX treatment, or by an indication bias: the IFX treatment could have been proposed to patients with less comorbidities. The observational design of this study does not enable to interpret this difference.

Lastly, all studies of administrative databases have a number of inherent limitations. The data quality is strongly influence by each institution’s collection and entry methods. For example, coding errors can lead to an inappropriate diagnosis. Heterogeneity in coding has been observed in various studies and various fields of medicine (Chantry and Bouvier-Colle, 2010; Gerbier et al., 2011; Bernier et al., 2012; Chantry et al., 2012; de Mey et al., 2012; Quantin et al., 2014; Guerra et al., 2015; Laplace et al., 2015; Pierron et al., 2015; Fermaut et al., 2016; Perozziello et al., 2018). Codification of the PMSI database is a complex procedure because of the large number of codes: more than 42,000 for ICD-10 (WHO, 2020), 13,000 for CCAM [L'Assurance Maladie (Ameli), 2020], and 2,000 for UCD [Agence Technique de l'information sur l'Hospitalisation (ATIH), 2020]. The French Technical Agency for Information on Hospital Care (*Agence Technique de l’Information sur l’Hospitalisation*) provides methodological guides for physicians and technicians responsible for coding. To the best of our knowledge, the coding validity for psychiatric events, inflammatory diseases or the drugs used to treat inflammatory diseases has not been studied.

### Perspectives

Our present results highlighted possible psychiatric adverse events associated with IFX therapy. These preliminary results must now be confirmed. It might be useful to link to other administrative databases, such as the French National Health Insurance Database (*Système National d* ***’*** *Information Interrégimes de l* ***’*** *Assurance Maladie*); the latter includes information on all dispensed prescription medicines (e.g. antidepressants, as a new outcome), and chronic medical conditions (as confounders). Moreover, it would be interesting to study the more recent data on IFX biosimilars and look at whether TNFα inhibitors have a class effect.

## Data Availability Statement

According to the French law, the authors are not allowed to share the data used for this study. Aggregated data are available on request to the corresponding author.

## Author Contributions

E-MT performed the study, analyzed the data, drafted the manuscript, and revised the manuscript. SG supervised the work (especially with regard to pharmacology), and critically revised the manuscript. EB supervised the work (especially with regard to statistics), and critically revised the manuscript. LC gave advice on the psychiatric events and pharmacology, and critically revised the manuscript. AA gave advice on the psychiatric events, and critically revised the manuscript. GB and J-BB gave advice on medical and methodological aspects of the work, and critically revised the manuscript. EC designed the study, managed, and extracted the data, supervised the study and the analysis, drafted the manuscript, and revised the manuscript. All the authors agree to be accountable for all aspects of the work, and provide approval for publication of the content.

## Conflict of Interest

The authors declare that the research was conducted in the absence of any commercial or financial relationships that could be construed as a potential conflict of interest.

## Correction note

A correction has been made to this article. Details can be found at: 10.3389/fphar.2025.1719490.

## Abbreviations list

CI, Confidence Interval; CCAM, *Classification commune des actes médicaux*; HR, Hazard Ratio; ICD-10, International Classification of Disease, 10^th^ edition; IFX, Infliximab; IQR, Interquartile Range; PMSI, *Programme de médicalisation des systèmes d’information*; SD, Standard Deviation; SPC, Summary of Product Characteristics; TNFα, Tumor Necrosis Factor alpha; UCD, *Unité commune de dispensation*.
