# Correction: Psychiatric adverse events associated with infliximab: a cohort study from the French nationwide discharge abstract database

**DOI:** 10.3389/fphar.2025.1719490

**Published:** 2025-10-22

**Authors:** Eve-Marie Thillard, Sophie Gautier, Evgeniya Babykina, Louise Carton, Ali Amad, Guillaume Bouzillé, Jean-Baptiste Beuscart, Emmanuel Chazard

**Affiliations:** ^1^ Univ. Lille, CHU Lille, ULR 2694, CERIM, Public Health Department, Lille, France; ^2^ Univ. Lille, Inserm, CHU Lille, UMR-S1172, Center for Pharmacovigilance, Lille, France; ^3^ Univ. Lille, Inserm, CHU Lille, UMR_S1172, Medical Pharmacology Department, Lille, France; ^4^ Univ. Lille, Inserm, CHU Lille, U1172 - LilNCog - Lille Neuroscience & Cognition, Lille, France; ^5^ University of Rennes, Inserm, CHU Rennes, UMR 1099 - LTSI, Rennes, France

**Keywords:** infliximab, adverse events, psychiatry, depression, database, pharmacoepidemiology, pharmacovigilance

**Author** Grégoire Ficheur was erroneously included as an author in the published article. The correct author list reads:

Eve-Marie Thillard, Sophie Gautier, Evgeniya Babykina, Louise Carton, Ali Amad, Guillaume Bouzillé, Jean-Baptiste Beuscart and Emmanuel Chazard.

The **Author Contributions Statement** has been corrected to read:

E-MT performed the study, analyzed the data, drafted the manuscript, and revised the manuscript. SG supervised the work (especially with regard to pharmacology), and critically revised the manuscript. EB supervised the work (especially with regard to statistics), and critically revised the manuscript. LC gave advice on the psychiatric events and pharmacology, and critically revised the manuscript. AA gave advice on the psychiatric events, and critically revised the manuscript. GB and J-BB gave advice on medical and methodological aspects of the work, and critically revised the manuscript. EC designed the study, managed, and extracted the data, supervised the study and the analysis, drafted the manuscript, and revised the manuscript. All the authors agree to be accountable for all aspects of the work, and provide approval for publication of the content.

The original article has been updated

