# Supplementary material for: Psychiatric Adverse Events Associated With Infliximab: A Cohort Study From the French Nationwide Discharge Abstract Database
Source: Front Pharmacol. 2020 Apr 22;11:513. doi: 10.3389/fphar.2020.00513 (PMC7188945; doi:10.3389/fphar.2020.00513)
Supplement: Supplementary file 1 [file Table_1.docx]

Supplementary Table

Supplementary Table 1: ICD-10 codes used to define adverse events

| **ICD-10 codes** | **Labels** | **Psychotic disorder** | **Manic episode** | **Depressive disorder** | **Self-harm attempt** |
| --- | --- | --- | --- | --- | --- |
| **F05** | Delirium, not induced by alcohol and other psychoactive substances | 1 | 0 | 0 | 0 |
| **F058** | Other delirium | 1 | 0 | 0 | 0 |
| **F059** | Delirium, unspecified | 1 | 0 | 0 | 0 |
| **F20** | Schizophrenia | 1 | 0 | 0 | 0 |
| **F200** | Paranoid schizophrenia | 1 | 0 | 0 | 0 |
| **F201** | Disorganized schizophrenia | 1 | 0 | 0 | 0 |
| **F202** | Catatonic schizophrenia | 1 | 0 | 0 | 0 |
| **F203** | Undifferentiated schizophrenia | 1 | 0 | 0 | 0 |
| **F204** | Post-schizophrenic depression | 0 | 0 | 1 | 0 |
| **F205** | Residual schizophrenia | 1 | 0 | 0 | 0 |
| **F206** | Simple schizophrenia | 1 | 0 | 0 | 0 |
| **F208** | Other schizophrenia | 1 | 0 | 0 | 0 |
| **F209** | Schizophrenia, unspecified | 1 | 0 | 0 | 0 |
| **F22** | Delusional disorders | 1 | 0 | 0 | 0 |
| **F23** | Brief psychotic disorder | 1 | 0 | 0 | 0 |
| **F24** | Shared psychotic disorder | 1 | 0 | 0 | 0 |
| **F25** | Schizoaffective disorders | 1 | 0 | 0 | 0 |
| **F250** | Schizoaffective disorder, manic type | 1 | 1 | 0 | 0 |
| **F251** | Schizoaffective disorder, depressive type | 1 | 0 | 1 | 0 |
| **F252** | Schizoaffective disorder, mixed type | 1 | 1 | 1 | 0 |
| **F258** | Other schizoaffective disorders | 1 | 1 | 1 | 0 |
| **F259** | Schizoaffective disorder, unspecified | 1 | 1 | 1 | 0 |
| **F28** | Other psychotic disorder not due to a substance or known physiological condition | 1 | 0 | 0 | 0 |
| **F29** | Unspecified psychosis not due to a substance or known physiological condition | 1 | 0 | 0 | 0 |
| **F30** | Manic episode | 0 | 1 | 0 | 0 |
| **F302** | Manic episode, severe with psychotic symptoms | 1 | 1 | 0 | 0 |
| **F308** | Other manic episodes | 0 | 1 | 0 | 0 |
| **F309** | Manic episode, unspecified | 0 | 1 | 0 | 0 |
| **F31** | Bipolar affective disorder | 0 | 1 | 1 | 0 |
| **F310** | Bipolar disorder, current episode hypomanic | 0 | 1 | 0 | 0 |
| **F311** | Bipolar affective disorder, current episode manic without psychotic symptoms | 0 | 1 | 0 | 0 |
| **F312** | Bipolar disorder, current episode manic severe with psychotic features | 1 | 1 | 0 | 0 |
| **F313** | Bipolar affective disorder, current episode mild or moderate depression | 0 | 0 | 1 | 0 |
| **F3130** | Bipolar disorder, current episode depressed, mild or moderate severity, unspecified | 0 | 0 | 1 | 0 |
| **F3131** | Bipolar disorder, current episode depressed, mild | 0 | 0 | 1 | 0 |
| **F314** | Bipolar disorder, current episode depressed, severe, without psychotic features | 0 | 0 | 1 | 0 |
| **F315** | Bipolar disorder, current episode depressed, severe, with psychotic features | 1 | 0 | 1 | 0 |
| **F316** | Bipolar affective disorder, current episode mixed | 0 | 1 | 1 | 0 |
| **F318** | Other bipolar affective disorders | 0 | 1 | 1 | 0 |
| **F319** | Bipolar disorder, unspecified | 0 | 1 | 1 | 0 |
| **F32** | Depressive episodes | 0 | 0 | 1 | 0 |
| **F320** | Major depressive disorder, single episode, mild | 0 | 0 | 1 | 0 |
| **F321** | Major depressive disorder, single episode, moderate | 0 | 0 | 1 | 0 |
| **F322** | Major depressive disorder, single episode, severe without psychotic features | 0 | 0 | 1 | 0 |
| **F323** | Major depressive disorder, single episode, severe with psychotic features | 1 | 0 | 1 | 0 |
| **F328** | Other depressive episodes | 0 | 0 | 1 | 0 |
| **F329** | Major depressive disorder, single episode, unspecified | 0 | 0 | 1 | 0 |
| **F33** | Recurrent depressive disorders | 0 | 0 | 1 | 0 |
| **F330** | Major depressive disorder, recurrent, mild | 0 | 0 | 1 | 0 |
| **F331** | Major depressive disorder, recurrent, moderate | 0 | 0 | 1 | 0 |
| **F332** | Major depressive disorder, recurrent severe without psychotic features | 0 | 0 | 1 | 0 |
| **F333** | Major depressive disorder, recurrent, severe with psychotic symptoms | 1 | 0 | 1 | 0 |
| **F338** | Other recurrent depressive disorders | 0 | 0 | 1 | 0 |
| **F339** | Major depressive disorder, recurrent, unspecified | 0 | 0 | 1 | 0 |
| **F340** | Cyclothymic disorder | 0 | 1 | 1 | 0 |
| **F341** | Dysthymic disorder | 0 | 0 | 1 | 0 |
| **F3810** | Recurrent brief depressive episodes | 0 | 0 | 1 | 0 |
| **F412** | Mixed anxiety and depressive disorders | 0 | 0 | 1 | 0 |
| **F4320** | Adjustment disorder, unspecified | 0 | 0 | 1 | 0 |
| **F4321** | Adjustment disorder with depressed mood | 0 | 0 | 1 | 0 |
| **F4322** | Adjustment disorder with anxiety | 0 | 0 | 1 | 0 |
| **F443** | Trance and possession disorders | 1 | 0 | 0 | 0 |
| **F920** | Depressive conduct disorder | 0 | 0 | 1 | 0 |
| **R440** | Auditory hallucinations | 1 | 0 | 0 | 0 |
| **R441** | Visual hallucinations | 1 | 0 | 0 | 0 |
| **R442** | Other hallucinations | 1 | 0 | 0 | 0 |
| **R443** | Hallucinations, unspecified | 1 | 0 | 0 | 0 |
| **X6** | Intentional self-poisoning | 0 | 0 | 0 | 1 |
| **X7** | Intentional self-harm | 0 | 0 | 0 | 1 |
| **X8** | Intentional self-harm | 0 | 0 | 0 | 1 |
